# Misregulation of ER-Golgi Vesicle Transport Induces ER Stress and Affects Seed Vigor and Stress Response

**DOI:** 10.3389/fpls.2018.00658

**Published:** 2018-05-18

**Authors:** Xiaonan Zhao, Xiufen Guo, Xiaofei Tang, Hailong Zhang, Mingjing Wang, Yun Kong, Xiaomeng Zhang, Zhenjie Zhao, Min Lv, Lixin Li

**Affiliations:** ^1^Key Laboratory of Saline-alkali Vegetation Ecology Restoration, Ministry of Education, Alkali Soil Natural Environmental Science Center, Northeast Forestry University, Harbin, China; ^2^Institute of Soybean Research, Heilongjiang Provincial Academy of Agricultural Sciences, Harbin, China

**Keywords:** ER-Golgi vesicle transport, MAG2 complex, seed vigor, ABA signaling, plant stress response

## Abstract

Seeds of higher plants accumulate numerous storage proteins to use as nitrogen resources for early plant development. Seed storage proteins (SSPs) are synthesized as large precursors on the rough endoplasmic reticulum (rER), and are delivered to protein storage vacuoles (PSVs) via vesicle transport, where they are processed to mature forms. We previously identified an *Arabidopsis* ER-localized tethering complex, MAG2 complex, which might be involved in Golgi to ER retrograde transport. The MAG2 complex is composed of 4 subunits, MAG2, MIP1, MIP2, and MIP3. Mutants with defective alleles for these subunits accumulated SSP precursors inside the ER lumen. Here, we report that the *mag2-1 mip3-1* and *mip2-1 mip3-1* double mutant have more serious vesicle transport defects than the *mag2-1*, *mip2-1*, and *mip3-1* single mutants, since they accumulate more SSP precursors than the corresponding single mutants, and ER stress is more severe than the single mutants. The *mag2-1 mip3-1* and *mip2-1 mip3-1* double mutants show growth and developmental defects rather than the single mutants. Both single and double mutant seeds are found to have lower protein content and decreased germinating vigor than wild type seeds. All the mutants are sensitive to abscisic acid (ABA) and salt stress, and exhibit alteration in ABA signaling pathway. Our study clarified that ER-Golgi vesicle transport affects seed vigor through controlling seed protein quality and content, as well as plant response to environmental stress via influencing ABA signaling pathway.

## Introduction

In higher plants, seeds are a critical life cycle stage, as they are important for the conservation of biodiversity and the persistence of species. Ideally, seeds should be tolerant to desiccation and harsh environments, and should be able to propagate after long term storage. Seed vigor is an important index of measuring seed quality. Seed vigor changes in response to aging, and is also sensitive to abiotic storage conditions such as moisture content, relative humidity, oxygen pressure, and temperature (Walters, 1998; Groot et al., 2012). There are four mechanisms affecting seed longevity: protection mechanisms, repair mechanisms, hormonal regulation of dormancy, and maternal effects.

Oxidation of cellular macromolecules is an important mechanism of seed aging (Harman and Mattick, 1976; Bailly, 2004). During storage, seed cell constituents are subjected to oxidative reactions, e.g., Amadori and Maillard reactions, lipid peroxidation, and protein carbonylation (Wilson and McDonald, 1986; Murthy and Sun, 2000; Arc et al., 2011). Accumulation of cellular oxidative damage due to seed aging or unsuitable storage conditions (such as high temperature and/or high humidity) thereby reduces seed vigor. Efficient antioxidant systems are therefore required to prevent excessive oxidation. These systems include enzymatic and non-enzymatic mechanisms. Enzymatic system includes superoxide dismutases, catalases, glutathione and ascorbate peroxidases, monodehydroascorbate, dehydroascorbate and glutathione reductases etc. (Bailly, 2004; Jeevan Kumar et al., 2015). Non-enzymatic mechanisms include SSPs and low molecular weight antioxidants such as tocopherol (vitamin E), ascorbate, and glutathione etc. (Sattler et al., 2004; Giurizatto et al., 2012; Sano et al., 2016). *Arabidopsis* VTE1 (a limiting factor in tocopherol biosynthesis), AtTIL (a temperature-induced lipocalin), and AtCHL (a chloroplastic lipocalin) are involved in lipid protection during seed aging (Havaux et al., 2005; Boca et al., 2014; Nguyen et al., 2015). SSPs are a major part of protecting system. They are very sensitive to oxidation. Generally, 12S globulin α-subunits are preferentially carbonylated to β-subunits in unaged seeds, while both types of the subunits are fully carbonylated in aged seeds (Job et al., 2005; Rajjou et al., 2008; Arc et al., 2011; Galland et al., 2012; Kalemba and Pukacka, 2014). *Arabidopsis* mutants with defective12S globulin genes showed reduced seed longevity, which implies that SSPs can play a key role in seed aging (Nguyen et al., 2015). In addition, there are several notable *Arabidopsis* seed mutants (e.g., *abi3*, *lec1*) with SSP content lower than those in wild type, display reduced seed longevity. HSPs are molecular chaperones that play an important role in protein folding and stability, as well as in protein protection against oxidative damage, which suggests their important role in seed longevity (Job et al., 2005; Prieto-Dapena et al., 2006). Another important strategy for seed survival is the deployment of repair systems during imbibition. Dry seeds are equipped with diverse protection mechanisms against various stresses. Nonetheless, after a prolonged period of storage, damage is accumulated in cell constituents which can be DNA, RNA, or proteins, all of which are essential for proper seed germination (Sano et al., 2016). Upon imbibition of dry seeds, the seed’s metabolic systems activated rapidly and the seeds must repair damage to initiate germination (Sano et al., 2016). The ability of seeds to repair this damage is in connection with seed longevity (Sano et al., 2016).

Seed longevity and dormancy are under the control of endogenous plant hormones (Baskin and Baskin, 2004; Long et al., 2015). It is reported that ABA is involved in both processes (Ooms et al., 1993). In ABA-deficient or insensitive mutants, seed longevity is closely associated with dormancy. Together with *LEC1* (*LEAFY COTYLEDON1)*, *LEC2* and *FUS3* (*FUSCA3*) *ABI3* is a dominating transcriptional regulator of seed maturation, and all *abi3*, *lec*, and *fus3* mutants exhibit decreased seed dormancy and longevity. These mutant seeds are intolerant to desiccation and fail to induce dormancy during the late maturation stage (Clerkx et al., 2004; North et al., 2010). *aba1-5* seeds showed remarkably reduced longevity, indicating that ABA has an important role in seed longevity (Clerkx et al., 2004). The combination of endogenous ABA deficiency with deficient ABA signaling in the *aba1-1 abi3-1* mutant resulted in a severe reduction in dormancy and longevity, corroborating the hypothesis that ABA controls both traits (Ooms et al., 1993). ABA receptors, including PYR, PYL, and RCAR proteins, bind to PP2Cs and inhibit their activity. PP2Cs act as negative regulators of the ABA pathway by dephosphorylating SnRK2 (sucrose non-fermenting 1-related subfamily 2) kinases (Cutler et al., 2010). Similarly, to *abi3*, the snrk2.2/2.3/2.6 triple mutant showed desiccation intolerance, absence of dormancy, and chlorophyll persistence, and was unable to complete seed development (Nakashima et al., 2009). Many genes encoding LEA and HSP proteins were downregulated in this mutant, including *AtEM1* and *AtEM6*, which are under control of *ABI5*, another phosphorylation target of SnRK2 kinases. EM homologs are correlated with the acquisition of desiccation tolerance and seed longevity (Chatelain et al., 2012).

Protein transport between cellular organelles including the ER, Golgi apparatus, endosomes, and vacuoles/lysosomes is mediated mainly by transport vesicles. COPII (Coat protein complex II) vesicles function in anterograde transport from the ER to the Golgi apparatus (Lee et al., 2004). COPI vesicles are responsible for retrograde transport from the Golgi apparatus to the ER for retrieval of ER-resident proteins, which are necessary for anterograde transport (Lee et al., 2004). Vesicle transport involves several essential steps, including budding, transport, tethering, docking, and fusion (Bonifacino and Glick, 2004). After budding from the donor membrane, vesicles are transported to their destination. The first interaction between vesicles and target membrane is mediated by tethering factors/complexes (Barlowe, 1997; Cao et al., 1998; Yu and Hughson, 2010). The vesicles then proceed to the docking step, where v-SNAREs (vesicle-soluble NSF [*N*-ethylmaleimide–sensitive fusion protein] attachment protein receptor) and t-SNAREs (target-membrane-SNAREs) combine to form a SNARE complex, thereafter promoting membrane fusion (Jahn and Scheller, 2006). Tethering factors are typically long or extended molecules, such as p115, GM130, and Uso1p (Whyte and Munro, 2002; Schmitt, 2010). Tethering complexes are divided into eight groups (Vukasinovic and Zarsky, 2016), the simplest complex among these is yeast Dsl1 complex, which consists of three subunits (Dsl1p, Sec39p, and Tip20p) (Kraynack et al., 2005). The Dsl1 complex is localized on the ER membrane through binding to the Ufe1p-Sec20p-Use1p SNARE complex and functions in retrograde transport from the Golgi apparatus to the ER (Andag and Schmitt, 2003; Kraynack et al., 2005; Ren et al., 2009; Tripathi et al., 2009). The Dsl1 complex is well conserved in higher eukaryotes, with homologs in mammals (ZW10-NAG-RINT-1) and *Arabidopsis thaliana* (MAG2-MIP1-MIP2-MIP3) (Xiao et al., 2001; Hirose et al., 2004; Arasaki et al., 2006; Li et al., 2006, 2013; Aoki et al., 2009; Schmitt, 2010). A homolog of the ZW10-NAG-RINT-1 complex-interacting SNARE complex in mammals has also been identified (i.e., syntaxin18-BNIP1-p31 complex) (Aoki et al., 2009; Schmitt, 2010). However, to date in *A. thaliana*, no homologous SNARE complex has yet been identified.

We previously found that the mutants deficient on the MGA2 complex subunits abnormally accumulate precursors of 2S albumins and 12S globulins, and develop numerous precursor-accumulating structures in the seed cells (i.e., MAG bodies, found within the ER lumen) (Li et al., 2006, 2013). It is suggested that storage proteins are prevented from exiting the ER in the MGA2 complex subunit-deficient mutants (Li et al., 2006, 2013). Although *mag2-1* and *mip3-1* seeds accumulate large amount of SSP precursors, there is no obvious abnormalities different from wild type during plant growth and development.

To elucidate the molecular machinery underlying MAG2-dependent protein transport pathways, we crossed *mag2-1* with *mip3-1* or *mip2-1* with *mip3-1* to generate *mag2-1 mip3-1* and *mip2-1 mip3-1* double mutants. We found that the double mutants exhibit growth and development defects, and more serious vesicle transport defects and ER stress than the single mutants. Furthermore, *mag2-1*, *mip2-1*, *mip3-1* single mutants and *mag2-1 mip3-1* and *mip2-1 mip3-1* double mutants have declined seed vigor because of decreased protein content and protein quality. Response of the mutant plants to environmental stress is altered, which might because of the alteration of ABA signaling.

## Materials and Methods

### Plant Materials and Growth Conditions

Wild type plants used in this study were *A. thaliana* ecotype Col-0. The *mag2-1*, *mip2-1*, and *mip3-1* mutants have been described previously (Li et al., 2006, 2013). Homozygous plants were obtained by PCR screening using gene-specific primers; primer sequences are shown in Supplementary Table S1. *Arabidopsis* seeds were surface-sterilized and sown either on soil or onto 0.8 or 1.2% agar with 1/2 Murashige and Skoog medium (PhytoTech, China) and 1% (w/v) sucrose. Plants were grown at 22°C under long-day (LD, 16 h light/8 h dark) conditions.

### RNA Extraction and RT-PCR and RT-PCR Analysis

Seedling RNA was isolated using an RNeasy kit (P4623, TIANGEN, China). Siliques were treated by the SDS method (Martin et al., 2005), followed by RNA extraction using an RNeasy kit. 0.5–1 μg of total RNA was reverse transcribed using the Revert Aid-TM First Strand cDNA Synthesis Kit (Fermentas, Burlington, ON, Canada). Semiquantitative RT-PCR was performed according to the manufacturer’s instructions. ACT2 was used as an endogenous control for RT-PCT, and ACT7 for qRT-PCR.

### Antibodies and Immunoblot Analysis

SDS–PAGE and immunoblot analysis were performed as described previously (Shimada et al., 2003). Antibody dilutions were as follows: anti-MAG2, 1:1000; anti-BiP (AS09 481, Agriser, Sweden), 1:2000; anti-ACT (AS13 2640, Agriser, Sweden), 1:1000; anti-12S, 1:20,000; anti-2S, 1:10,000; anti-dinitrophenol (ab6306, abcam), 1:1000; anti-ABI5 (ab98831, abcam), 1:2000; anti-tubulin A (R0267-1a, Abiocode), 1:2500. The dilution of horseradish peroxidase-conjugated rabbit antibodies raised against rabbit IgG (ZB2301, ZSGB-BIO, China) was 1:5000. Signals were detected using an enhanced chemiluminescence (ECL) detection system (LAS-4000, FYJIFILM, Japan).

### Detection of Protein Carbonylation

Quantification of carbonylation: A Plant PC ELISA Kit (ml092011-2, Mlbio, China) was used for quantification of seed protein carbonylation. Quantification of all samples was entrusted to Mlbio. A standard curve was generated by plotting the average O.D. (450 nm) obtained for each of the six standard concentrations on the vertical (X) axis, with the corresponding concentration on the horizontal (Y) axis.

Immunodetection of carbonylated proteins: The carbonyl groups in proteins were analyzed by immunodetection of protein-bound DNP after derivatization with the corresponding hydrazine, as described (Talent et al., 1998; Korolainen et al., 2002; Job et al., 2005) with slight modifications. One microgram of seeds were grinded with 100 μl of protein extraction buffer [10 μg/mL; 0.1 M Tris-HCl (pH8.0), 0.9 M Sucrose, 10 mM EDTA, 0.4% β-mercaptoethanol, 0.8% SDS]. Following dialysis (Merck), four volumes of 10 mmol DNPH (Sigma)/2 mol HCl were added. Samples were agitated for 30 min at room temperature, and five volumes of 20/80 ice-cold TCA-acetone containing 1 mmol DTT were added to each sample. The samples were centrifuged for 15 min at 15,000*g* at 4°C. The precipitated protein was then washed three times with 1 mL of 1:1 (v/v) ethanol:ethyl acetate and resolubilized in the thiourea/urea lysis buffer [7 M Urea, 2 M Thiourea, 2% (v/v) Triton X-100, 20 mM DTT, Protease inhibitor]. Proteins were separated by SDS-PAGE and transferred to PVDF (Merck, Immobilon-P^SQ^ Transfer Membranes) using standard procedures. Blots were rinsed twice for 5 min in 50 mmol Tris-HCl, 150 mmol NaCl, pH 7.5 (TBS), then incubated for 1 h at 25°C in Blocking Solution (5% Skim milk). After incubation for 1 h with rabbit anti-DNP antibodies (abcam) in TBS, blots were washed four times with TBS containing 0.05% (v/v) Tween 20 and incubated for 1 h in peroxidase-conjugated goat anti-rabbit IgG (ZB2301, ZSGB-BIO, China). Bound antibodies were visualized by using the ECL kit (Thermo).

### Accession Numbers

GenBank/EMBL accession numbers and *Arabidopsis* Genome Initiative locus identifiers for the genes mentioned in this article are as follows: MAG2, At3g47700.1; MIP1, At2g32900; MIP2, At5g24350; MIP3, At2g42700.

## Results

### Generation of ER-Localized Tether-Defective Double Mutants

**FIGURE 1:**
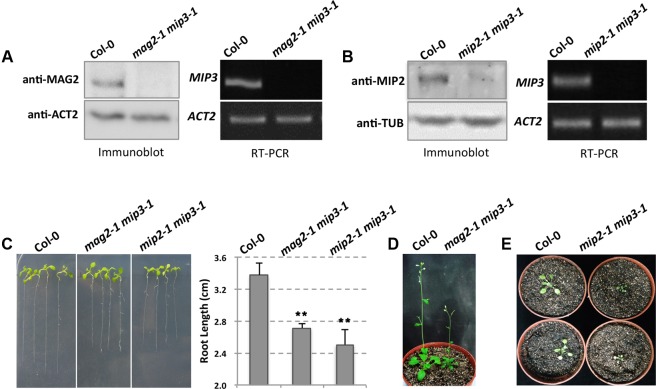
The *mag2-1 mip3-1* double mutant exhibits dwarf phenotype. **(A)** Immunoblot of maturing seeds from the wild type (Col-0, the same below) and *mag2-1 mip3-1* double mutant with anti-MAG2 antibody (left panel). Right panel indicates expression level of *MIP3* in 9-day-old seedlings of wild type and *mag2-1 mip3-1* double mutant. RT-PCR was performed using *MIP3* specific primers (Li et al., 2013). **(B)** Immunoblot of 7-day-old seedlings from the wild type and *mip2-1 mip3-1* double mutant with anti-MIP2 antibody (left panel). Right panel indicates expression level of *MIP3* in 7-day-old seedlings of wild type and *mip2-1 mip3-1* double mutant. **(C)** 7-day-old seedlings of wild type, *mag2-1 mip3-1* and *mip2-1 mip3-1* double mutants (left panel) and root length statistics (right panel). *n* = 30, two independent experiments per sample. **(D)** 41-day-old plants of wild type and *mag2-1 mip3-1*. **(E)** 21-day-old plants of wild type and *mip2-1 mip3-1*.

To clarify how the Golgi-to-ER retrograde pathway affects SSP transport, we generated a *mag2-1 mip3-1* double mutant by crossing *mag2-1* and *mip3-1* single mutants, which were developed in previous study (Li et al., 2006, 2013). It was previously reported that MAG2 protein is absent in *mag2-1*, and *MIP3* mRNA was not detected in *mip3-1*, which indicate these two mutants are null (Li et al., 2006). Immunoblot result indicates that MAG2 is depleted in *mag2-1 mip3-1*. Similarly, RT-PCR result indicates that *MIP3* is not expressed in *mag2-1 mip3-1* (**Figure 1A**).

Different from single mutants *mag2-1* and *mip3-1* which don’t have obvious phenotypes, the double mutant *mag2-1 mip3-1* plants have abnormal phenotypes. Seven-day-old seedlings root length of *mag2-1 mip3-1* is shorter than that of wild type (**Figure 1C**). In addition, *mag2-1 mip3-1* plants grow poorly and have small rosette leaves with short petioles, the plant height is much shorter than that of wild type (**Figure 1D** and Supplementary Figure S1). These abnormalities indicate that the MAG2 and MIP3 protein simultaneous deficiency affects the development of vegetative organs.

Recently, we isolated *mip2-1* homozygous which we couldn’t obtain in previous study (Li et al., 2013), and generated another double mutant *mip2-1 mip3-1*. In *mip2-1 mip3-1*, MIP2 protein level is very low, indicating that *MIP2* gene is down-regulated (**Figure 1B**). This is identical to the level in *mip2-1* single mutant (Supplementary Figure S4). *mip2-1 mip3-1* also exhibits shorter root and delayed development (**Figures 1C,E**).

### Vesicle Trafficking Deficient in Mutant Seed Cells

**FIGURE 2:**
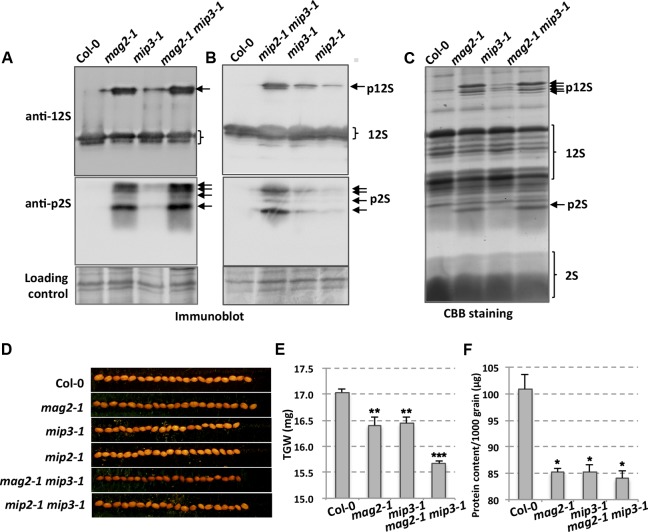
Defects in storage protein trafficking for the *mag2-1*, *mip3-1* and *mag2-1 mip3-1* mutants. **(A,B)** Immunoblot of dry seeds of wild-type and the mutants with anti-12S and anti-p2S antibodies. Arrows indicate SSP precursors. p12S, pro12S globulin; p2S, pro2S albumin; 12S, 12S globulin; 2S, 2S albumin. **(C)** Seed protein profile. Coomassie brilliant blue (CBB) staining of an SDS–PAGE gel for seed proteins. Abnormally accumulated SSP precursors are indicated by arrows. **(D)** Seed grains of wild type and the mutants. **(E)** Thousand-grain-weight (TGW) for wild type and the mutant seeds. **(F)** Protein content of one thousand grains is measured by BCA Protein Quantification Kit (ml410021, Mlbio, China).

Our previous work showed that MAG2, MIP2, and MIP3 are involved in vesicle trafficking between the ER and the Golgi apparatus. When MAG2, MIP2 or MIP3 is defective, the exit efficiency of the SSP precursors from the ER is disrupted. In *mag2-1*, *mip2-1* and *mip3-1* mutant seeds, large amounts of SSP precursors were accumulated (**Figures 2A–C**) (Li et al., 2006, 2013). Examinations of seeds from the double mutants *mag2-1 mip3-1* and *mip2-1 mip3-1* found even higher accumulation of SSP precursors, suggesting that SSP transport is seriously disrupted and SSPs have severe difficulties escaping from the ER when MAG2 and MIP3 or MIP2 and MIP3 are simultaneously deficient. The mutant seeds look smaller than wild type seeds (**Figure 2D**). To see if the defects affect seed total protein, we measured thousand-grain weight (TGW) and protein content of *mag2-1*, *mip3-1* and *mag2-1 mip3-1*. Both the TGW and the protein content of the single mutants and double mutant are lower than the wild type, and that of double mutant are lower than the single mutants (**Figures 2E,F**).

### ER Stress Is Induced in Mutant Seeds

**FIGURE 3:**
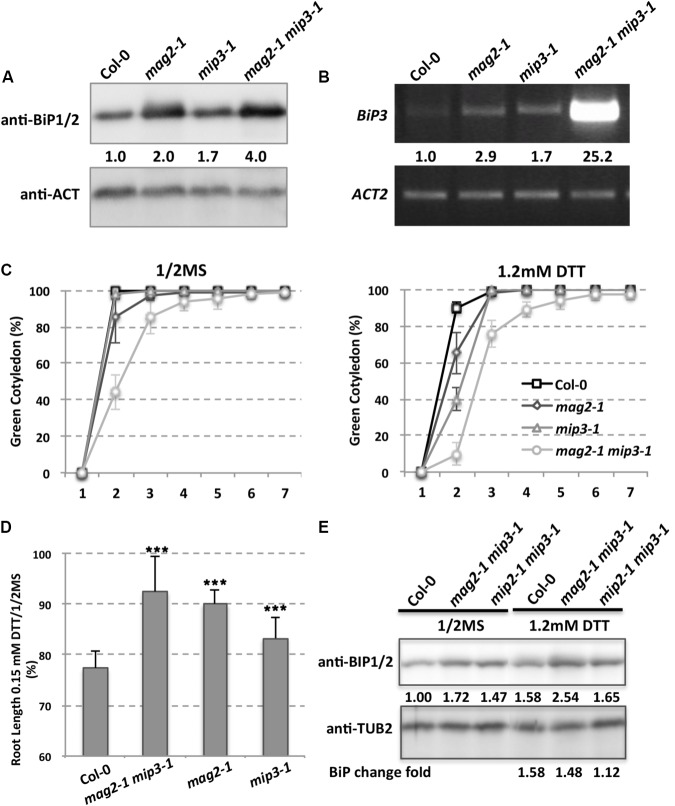
ER stress is induced in the mutants. **(A)** Immunoblot of dry seeds of wild type, *mag2-1*, *mip3-1* and *mag2-1 mip3-1*, with anti-BiP1/2 antibodies. Statistics for BiP1/2 protein accumulation in seeds are shown in numbers below the bands BiP/control (ACT or TUB, actin or tubulin) band concentration, measured by ImageJ, the same below). Three independent experiments per sample. **(B)** *BiP3* expression was detected by RT-PCR on 9-day-old seedlings using *BiP3* specific primers. Statistics for *BIP3* expression level are shown in numbers below the bands. *n* = 3. Three independent experiments per sample. **(C)** Quantitative analysis of germination ratio (Green cotyledon ratio, the same below) of wild type, *mag2-1*, *mip3-1* and *mag2-1 mip3-1* on 1/2MS medium (left) or 1/2MS medium with 1.2 mM DTT (right). *n* = 72, three independent experiments per sample. **(D)** Root length change fold (treated/non-treated) of 7-old-seedlings of wild type, *mag2-1 mip3-1* and *mip2-1 mip3-1* on 1/2MS medium (non-treated) and 1/2MS medium with 0.15 mM DTT (treated). **(E)** Immunoblot on DTT treated 7-day-old seedlings of wild type, *mag2-1*, *mip3-1* and *mag2-1 mip3-1* on 1/2MS medium (left panel) or 1/2MS medium with 1.2 mM DTT (right panel) with anti-BiP1/2 antibody. Statistics of BiP1/2 protein level are shown below the bands. Change fold (treated/non-treated, the same below) is indicated in the lowest panel.

Due to defects on protein exit from the ER, SSP precursors accumulated inside the ER lumen in the mutant seed cells, which therefore induces ER stress. ER chaperones are known to increase when cells suffer from ER stress. To this end, *A. thaliana* has ubiquitously expressed genes *BiP1*, *BiP2*, and *BiP3*. *BiP3* is induced only under ER stress or abiotic stress conditions (Koizumi, 1996; Martinez and Chrispeels, 2003; Iwata and Koizumi, 2005; Liu et al., 2007; Iwata et al., 2008, 2010a,b; Tajima et al., 2008; Liu and Howell, 2010). *BiP1* and *BiP2* are nearly 99% identical while *BiP3* has an identity of about 80% relative to *BiP1* and *BiP2* (Noh et al., 2003). Previous work showed that *BiP1* and *BiP2* were abnormally accumulated in *mag2* seeds (Li et al., 2006), and therefore we checked *BiP1/2* accumulation in *mag2-1*, *mip3-1* and *mag2-1 mip3-1* mutant seeds. As expected, *BiP1/2* accumulation in the single and double mutant seeds was higher than in wild type seeds (**Figure 3A**), indicating a higher degree of ER stress in the mutants. In *mag2-1 mip3-1* double mutant seeds, the concentration of *BiP1/2* showed a fourfold increase, which was much higher than the increases found in the single mutants, suggesting that simultaneous deficiency at the *MAG2* and *MIP3* loci causes severe ER stress. For the stress-specific-induced *BiP3*, in the single and double mutant 7-day-old seedlings, it is largely upregulated compared to the wild type. Notably, the *mag2-1 mip3-1* double mutant shows significant increase in *BiP3* expression (**Figure 3B**), suggesting severe ER stress is induced in these mutants. Even in *mag2-1 mip3-1* and *mip2-1 mip3-1* double mutant plants, BiP1/2 protein level is higher than that in wild type (**Figure 3E** left panel). This should be due to the serious disruption of ER-Golgi vesicle transport in the mutants.

To see how the mutants response to exogenous ER stress, we performed dithiothreitol (DTT) treatment which can cause ER stress (Braakman et al., 1992). Under 1.2 mM DTT treatment, the germination ratio of 7-DAG seedlings of double mutant decreased compared with untreated ones, moreover, germination of the single and double mutants were delayed at the beginning days (**Figure 3C**). The root lengths of 7-day-old seedlings of both wild type and mutants were shorter under DTT treatment than non-treatment, but the root length change (treatment/non-treatment) in wild type was larger than those of the mutants, suggesting the mutants are insensitive to DTT stress (**Figure 3D**). We also checked BiP1/2 protein level under DTT treatment, BiP1/2 protein change fold (treated/non-treated) in *mag2-1 mip3-1* and *mip2-1 mip3-1* were lower than that in wild type, suggesting that the mutants are insensitive to DTT treatment.

### Seed Longevity Is Reduced in Mutants

**FIGURE 4:**
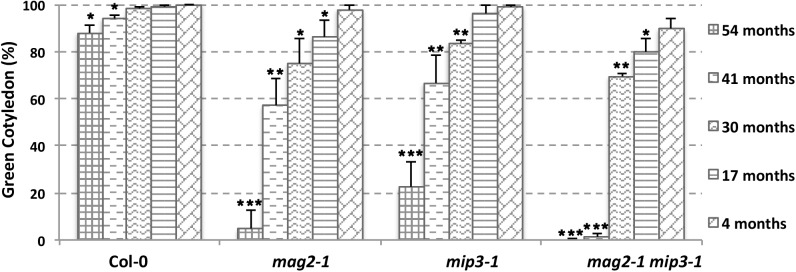
Vigor of aging mutant seeds decreased rapidly. Germination ratio statistics for seeds from different storage time on the 7th day after germination (DAG). *n* = 50, three independent experiments per sample. The seeds used in this experiment were collected from 2013 to 2017.

The abnormal accumulation of SSP precursors and the presence of ER stress may cause a reduction in seed protein quality, which may have secondary effects on seed longevity. To determine whether this was the case, we checked germination vigor of mutant seeds. We selected seeds harvested during the last 5 years. As shown in **Figure 4**, all mutant seeds showed a decreased germination ratio (green cotyledon ratio, the same below) as storage time extended. Remarkably, the *mag2-1 mip3-1* double mutant showed a dramatically lower germination ratio after 3 years of storage (41 and 54 months). These results are identical to another statistical analysis performed 4 years ago (Supplementary Figure S2). These tendencies in germination ratio are inversely proportional to SSP precursor accumulation level in the mutant seeds. We believe that these results constitute direct evidence that the quality of seed proteins affects seed vigor.

### SSPs Function as Oxidation Buffer on Seed Longevity

**FIGURE 5:**
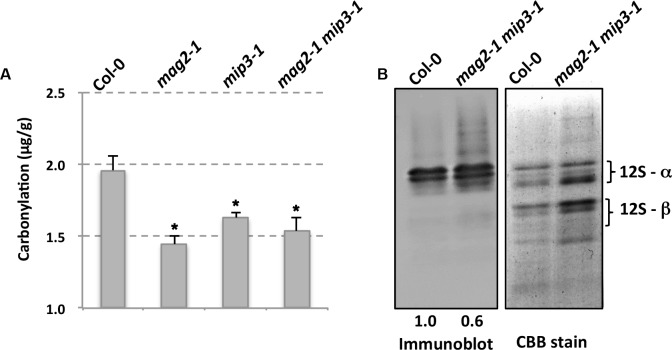
Mutant seeds have lower protein carbonylation. **(A)** Quantification of seed protein carbonylation of wild type and mutants. **(B)** Carbonylated proteins were detected by immunodetection of protein-bound DNP after derivatization with hydrazine with anti-Dinitrophenol antibody (left panel). Right panel is CBB staining of a SDS–PAGE gel for total protein extracts from wild type and mutant seeds. Statistics of the bands are shown in numbers below the bands.

It is mentioned that SSPs buffer the seed cells from oxidative stress, thus protecting important proteins which are required for seed germination and seedling formation (Nguyen et al., 2015). It should be noticed that these functional protecting SSPs are the mature forms which are processed after transported to the PSVs. Protein carbonylation is a widely used marker of protein oxidation (Ballesteros et al., 2001; Das et al., 2001; Mostertz and Hecker, 2003; Johansson et al., 2004). Since seed longevity is affected in the single and double mutants, we determined seed protein carbonylation. Quantification of seed protein carbonylation indicates that the carbonylation levels in all mutant seeds were decreased compared to that in wild type seeds (**Figure 5**). SSP carbonylation is a protecting mechanism against oxidation, lower carbonylation level may be the main reason for the lower germination vigor of the mutant seeds. This point is also mentioned in previous study (Job et al., 2005; Prieto-Dapena et al., 2006).

### ABA Signaling Pathway Is Influenced in the Mutants

**FIGURE 6:**
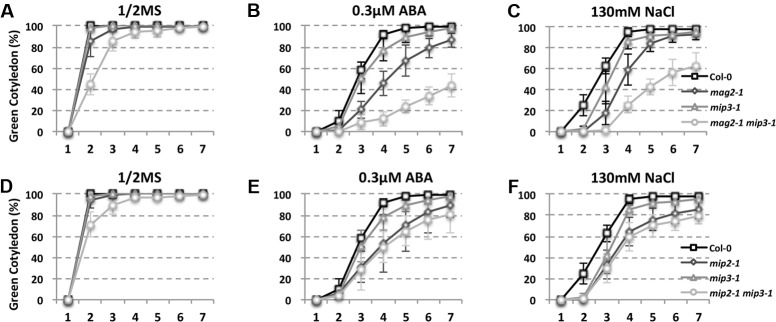
ABA and NaCl treatments result in a greater germination ratio decline in mutants than in wild type. Quantitative analysis of germination ratio of wild type and the mutants on 1/2MS medium **(A,D)**, 1/2MS medium with 0.3 μM ABA **(B,E)** or with 130 mM NaCl **(C,F)**. *n* = 50, three independent experiments per sample.

**FIGURE 7:**
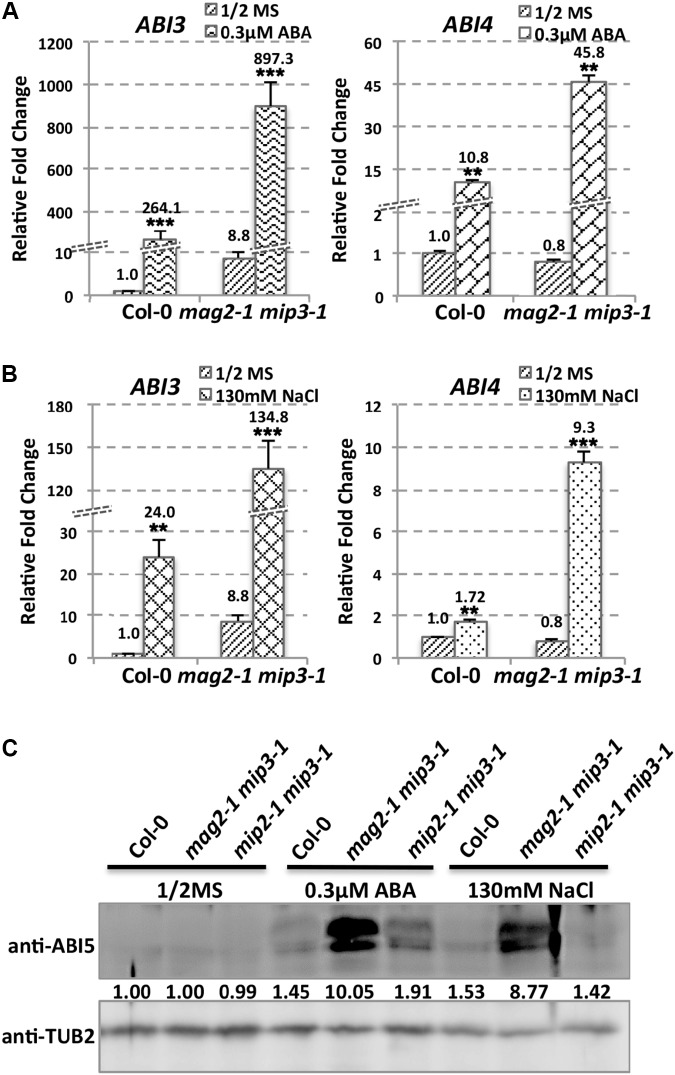
*ABI3* and *ABI4* expression level altered in mutants. *ABI3* and *ABI4* expression level in wild type and *mag2-1 mip3-1* under ABA treatment **(A)** or under NaCl treatment **(B)** were determined by qRT-PCR on 10-day-old seedlings. For qRT-PCR, three repeats per experiment, three experiments per sample. **(C)** Immunoblot on the ABA and NaCl treated 10-day-old seedlings with anti-ABI5 antibody. Statistics of ABI5 protein level are shown below the bands.

**FIGURE 8:**
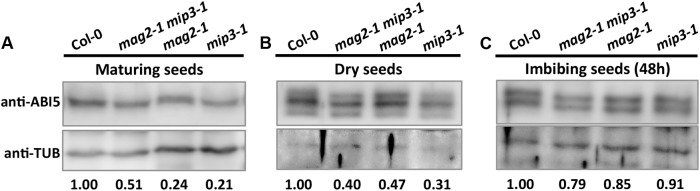
ABI5 protein accumulation level altered in mutant seeds. Immunoblot with anti-ABI5 antibody on maturing seeds from the No. 2 and No. 3 siliques below the inflorescences **(A)**, dry seeds **(B)** and imbibing seeds after 48 h imbibition **(C)**. Lower panels are statistics of upper panels.

As mentioned above, ABA is known to control both seed dormancy and longevity (Sano et al., 2016). In addition, ABA plays a crucial role in the plant’s response to abiotic (drought, salinity, cold, and hypoxia) and biotic stresses. Therefore, we investigated the effects of exogenous ABA on seed germination using 5-month storage seeds. As shown in **Figures 6B,E**, ABA treatment resulted in a greater decline in the germination ratio in the single and double mutants than in the wild type. The greatest decline was in the *mag2-1 mip3-1* double mutant, where the germination ratio decreased by about 43% (compared to 95% in the wild type). NaCl treatment also resulted in a large reduction of germination rate in the mutants (**Figure 6C**). Above results indicate that these ER-Golgi vesicle trafficking deficient mutants were sensitive to ABA and salt stresses. ABA may control water relations and H_2_O_2_ accumulation via ABI3 modulation of aquaporins, thereby contributing to seed longevity (Sano et al., 2016), and ABI5 is genetically downstream of ABI3 (Finkelstein and Lynch, 2000; Lopez-Molina et al., 2002), ABI4 is a key regulator of seed dormancy, germination and post-germination growth in the ABA signaling pathway (Soderman et al., 2000; Shkolnik-Inbar and Bar-Zvi, 2010) ABI4 also plays an essential role in ABA and cytokinin signaling cross talk (Shu et al., 2013; Huang et al., 2017). Thus, we detected *ABI3*, *ABI4* and *ABI5* expression levels. In normal condition, *ABI3* expression in *mag2-1 mip3-1* and *mip2-1 mip3-1* is higher than that in wild type, but *ABI4* expression level and ABI5 protein accumulation level have no big difference between the double mutants and wild type (**Figure 7** and Supplementary Figure S3). Under ABA and NaCl treatments, *ABI3* and *ABI4* expression all increased in wild type and the two double mutants (**Figures 7A,B** and Supplementary Figures S3A,B). Under ABA and NaCl treatment, *ABI3* expression increasing folds (treated/untreated) in *mag2-1 mip3-1* were lower than those in wild type. This is because that the original (untreated) *ABI3* expression level in *mag2-1 mip3-1* is much higher than that in wild type. The changing range of *ABI3* expression in *mip2-1 mip3-1*, of *ABI4* expression in both *mag2-1 mip3-1* and *mip2-1 mip3-1* were higher than those in wild type. In *mag2-1 mip3-1*, ABI5 protein accumulation level increased significantly under ABA and NaCl treatments, but in *mip2-1 mip3-1*, ABI5 protein only increased under ABA treatment (**Figure 7C**). While, in maturing seeds, dry seeds and imbibing seeds, ABI5 protein accumulation level in the single and double mutants is lower than that in wild type (**Figure 8**). All these results suggest that ABA signaling pathway is affected in the ER-Golgi vesicle transport deficient mutants.

## Discussion

In eukaryotic cells, vesicle transport and recycling are basics for cell activities such as maintenance of organelle functions, cellular homeostasis, cytokinesis, adaptation to the environment and intercellular communication (Surpin and Raikhel, 2004; Ebine et al., 2008).

### ER-Tethering Complex, MAG2 Complex Is Involved in Plant Growth and Development

Our previous work showed that in *Arabidopsis*, MAG2 and three MIP proteins form a stable complex on the ER membrane, and are required for efficient exit of SSP precursors from the ER. The MAG2 complex may facilitate retrograde transport from the Golgi apparatus to the ER (Li et al., 2006, 2013). The MAG2- and MIP-deficient mutants abnormally accumulate SSP precursors in the ER and form a novel cell structure-MAG2 Body, within the ER lumen. Although there are such drastic abnormalities in the seed cells, we didn’t observe obvious abnormalities in vegetative organs and plant growth and developmental stages in *mag2* and *mip* mutants. This might because that defects on a single subunit of the MAG2 complex are functionally complemented by other existing subunits. But when MAG2 and MIP3 or MAG2 and MIP2 simultaneously deficient, the remaining subunits are incompetent to conduct the transport along with plant development stages, and thus the double mutants *mag2-1 mip3-1* and *mip2-1 mip3-1* showed obvious abnormalities in growth and development. Supplementary Figure S4 shows that the subunit accumulation levels are influenced in the mutants. This reflects that when one or more subunits of the complex is/are defected, the existing subunits adjust their accumulating level to make the plants adapted to the defects.

### Defects in ER-Golgi Vesicle Transport Affect Seed Longevity

In this study, we showed that double mutant *mag2-1 mip3-1* and *mip2-1 mip3-1* have serious disruption on ER-Golgi vesicle transport (**Figure 2**) and experiences severe ER stress than the single mutants (**Figure 3**), and exhibits growth and development defects (**Figure 1**). Since the SSP precursors cannot be delivered to vacuoles to be processed to the normal mature forms, the quality of seed proteins in the single mutants and double mutants are reduced. It is reported that the mature forms of 12S globulins, one of the main SSPs in *Arabidopsis*, are carbonylated primarily than other seed proteins. Therefore, it is proposed that SSPs are the most important mechanism for protecting other cell components and cell structures from oxidative stress. Due to their high affinity of oxidation, SSPs function as an oxidation buffer that helps the embryo survive seed storage (Nguyen et al., 2015). In the mutant seeds, protein oxidation namely carbonylation is lower than wild type (**Figure 5**). This might due to two reasons. One is that total protein content is decreased in the mutant seeds (**Figures 2D,F**), another reason is that accumulation of SSP precursors relatively reduce the SSP mature forms (**Figure 2**). Thus, reduced protein carbonylation may not be sufficient to defend the proteins which are important for embryo survival against oxidation, therefore result in the loss of seed vigor (**Figure 4**).

On the other hand, in the single and double mutants, ABI5 protein accumulation level in maturing seeds, dry seeds and imbibing seeds is lower than that in wild type (**Figure 8**), suggesting lower ABA level during seed maturation and imbibition in the mutants. As the major downstream component of ABA signaling, ABI3 is a main regulator of seed dormancy and germination (Bentsink and Koornneef, 2008). On the contrary, ABI5 has no effect on seed dormancy (Finkelstein, 1994), it represses seed germination (Piskurewicz et al., 2008; Kanai et al., 2010). In line with their claims, high endogenous ABA level stimulates ABI5 protein accumulation and activity which results in inhibition of seeds germination. But this is not the case for *mag2-1, mip3-1* and *mag2-1 mip3-1* mutants. Although these mutants have less ABI5 protein accumulation in the seeds, they have lower germination ratio. This indicates that ABA level is not the right reason causing the low germination. In another word, the alteration of ABA signaling pathway in the mutants is not the reason responsible for the lower seed vigor. Taken together, we reach a conclusion that defects on ER-Golgi vesicle transport affect seed longevity by influencing seed protein quality and content.

### ABA Signaling Pathways Are Affected by Mutations Rendering ER-Golgi Vesicle Transport Defective

During seed maturation, endogenous ABA accumulated in the seeds, inducing and maintaining seed dormancy and thus preventing vivipary. Endogenous ABA level and ABA signaling positively regulate seed dormancy and therefore negatively regulate seed germination (Vaistij et al., 2013). In addition to ABA biogenesis, the ABA-signaling-dependent pathway also affects seed dormancy. ABI3 positively regulates seed dormancy and germination (Bentsink and Koornneef, 2008), while, ABI5 negatively regulates seed germination (Piskurewicz et al., 2008; Kanai et al., 2010), suggesting the distinct signaling pathways for ABA-mediated seed dormancy and ABA-inhibited seed germination. ABA and gibberellin (GA) antagonistically regulate diverse plant developmental processes including seed dormancy and germination, root development, and flowering time control, and thus the optimal balance between ABA and GA is essential for plant growth and development (Shu et al., 2018). *Arabidopsis* ABI4 plays key roles in ABA and GA antagonism (Shu et al., 2013, 2016a,b,c).

In *mag2-1 mip3-1* and *mip2-1 mip3-1* mutants, ABI3 and ABI5 expression have different manner from wild type under ABA and salt treatments or during seed maturation and imbibition (**Figures 7, 8**), and the mutant seeds are sensitive to ABA and salt stress (**Figure 6**). It was recently reported by Zhao et al. (2013) that MAG2 is involved in ABA-mediated response to abiotic stress. All these suggest that ABA signaling pathways are altered due to the defects on ER-Golgi vesicle trafficking. It is considerable that ABI5 phosphorylation type (upper band) significantly changed under ABA treatment and NaCl treatment in *mag2-1 mip3-1* (**Figure 7C**). It is reported that PP6 (FyPP1/3) proteins act antagonistically with SnRK2 kinases to regulate ABI5 phosphorylation and ABA responses (Dai et al., 2013). Protein posttranslational modifications (PTM), including protein phosphorylation/dephosphorylation, ubiquitination, and sumoylation, regulate ABI5 signaling (Lopez-Molina et al., 2002; Lois et al., 2003; Ma et al., 2009; Miura et al., 2009; Cutler et al., 2010; Miura and Hasegawa, 2010; Hu and Yu, 2014; Yu et al., 2015). *Arabidopsis* RAV1 transcription factor plays an important role in ABA signaling by modulating the expression of *ABI3*, *ABI4*, and *ABI5*, and that its activity is negatively affected by SnRK2s (Feng et al., 2014). To the end, which process(es) during the ABA signaling pathway is/are affected in the mutants is an interesting topic and need further study. And the ABA and GA antagonism disequilibrium in the mutants is also a challenging issue.

### Severe ER Stress Induced in the Mutants Affect Plant Response to Environment

We found that serious ER stress is induced in our mutants, and the plants are insensitive to DTT treatment (**Figures 3D,E**). There are two possibilities, one is that ER stress induced UPR (unfold protein response) pathway might be affected, another possibility is that *mag2-1 mip3-1* and *mip2-1 mip3-1* themselves have severe endogenous ER stress, this results in insensitivities of the mutants to exogenous ER stress. To verify the assumption, we checked expression level of UPR pathway markers. The types of UPR recognized in plants are the ATF6 homolog and IRE1 pathways. IRE1 monitors ER homeostasis through an ER luminal stress sensing domain and triggers UPR (Hetz and Glimcher, 2009; Hetz et al., 2011). Two *Arabidopsis* IRE1 isoforms IRE1A or IRE1B share partially overlapping function during the UPR (Nagashima et al., 2011; Chen and Brandizzi, 2012). GAAP1/GAAP3 play roles in the negative regulation of IRE1 activity (Guo et al., 2018). AtbZIP28 is homologs for ATF6 (Liu et al., 2007). As Supplementary Figures S5A,B indicated, IRE1A and bZIP28 decreased more in the double mutants than in wild type, and IRE1B increased in the double mutants in contrary to that in wild type, suggesting the UPR pathway is affected in the mutants. On the other hand, we can’t exclude the possibility that endogenous ER stress *in mag2-1 mip3-1* and *mip2-1 mip3-1* reduce sensitivities to exogenous ER stress. Maybe these two possibilities simultaneously exist.

Since the ER stress and ABA signaling are closely related with the phenotypes of *mag2-1 mip3-1* and *mip2-1 mip3-1*, we checked BiP1/2 protein level under ABA and salt treatments. We found that BiP1/2 protein change fold increased under these stresses in wild type and double mutants. While, in *mag2-1 mip3-1*, BiP1/2 protein change fold was higher than that in wild type, but in *mip2-1 mip3-1*, BiP1/2 protein change fold was lower than that in wild type. This difference supports role division between the subunits in MAG2 complex (Supplementary Figure S5E). We also checked ABA signaling pathway under DTT treatment. As Supplementary Figures S5C,D indicated, *ABI3* and *ABI4* expression decreased under DTT treatment in wild type and double mutants, except for *ABI4* in *mag2-1 mip3-1*. In *mag2-1 mip3-1*, *ABI4* expression increased. *ABI3* expression in *mip2-1 mip3-1*, *ABI3* and *ABI4* expression in *mip2-1 mip3-1* decreased more than those in wild type (Supplementary Figure S5E). Similarly, ABI5 protein level in *mag2-1 mip3-1* increased, but in *mip2-1 mip3-1* decreased (Supplementary Figure S5F). Above results suggest that in *mag2-1 mip3-1* and *mip2-1 mip3-1*, ABA signaling pathway is also affected under exogenous ER stress. In another word, plant response to environment is affected in the mutants.

### Limitations and Future Applications

The endoplasmic reticulum is the starting point for the secretory pathway and can be viewed as a biosynthetic hub within the cell. The ER has diverse functions, including synthesis of secretory proteins, membrane proteins as well as lipids, protein folding, posttranslational modification, export of proteins, and calcium storage. Various interior and exterior stimuli factors, including reduction of disulfide bonds, interference of glycosylation, calcium depletion from the ER lumen, and block of protein transport from the ER to the Golgi apparatus disturb ER functions and induce ER stress (Uemura et al., 2009). ER stress has been suggested to be involved in various disorders (Kadowaki et al., 2004; Marciniak and Ron, 2006; Yoshida, 2007). In the ER-Golgi vesicle transport deficient mutants, the disorders are reflected in incapable of exit of the SSP precursors, alteration of ABA signaling and defects on plant growth and development. This raises a question about what is the relationship between vesicle trafficking and ABA signaling, and how defects on ER-Golgi vesicle trafficking causes alteration of ABA signaling. These could be a challenging cross talk between vesicle trafficking and plant responses to environmental stresses.

## Author Contributions

LL the corresponding author of the manuscript and figures, designed the research with assistance of XNZ. XNZ and LL analyzed the data. XNZ conducted all the experiments with help of XG, XT, MW, YK, HZ, XMZ, ZZ, and ML. LL and XNZ wrote the manuscript. LL edited the manuscript. All authors discussed the results and commented on the manuscript. All authors read and approved the final manuscript.

## Conflict of Interest Statement

The authors declare that the research was conducted in the absence of any commercial or financial relationships that could be construed as a potential conflict of interest.

## References

[B1] Andag U. Schmitt H. D. (2003). Dsl1p, an essential component of the Golgi-endoplasmic reticulum retrieval system in yeast, uses the same sequence motif to interact with different subunits of the COPI vesicle coat. 278 51722–51734. 10.1074/jbc.M308740200 14504276

[B2] Aoki T. Ichimura S. Itoh A. Kuramoto M. Shinkawa T. Isobe T. (2009). Identification of the neuroblastoma-amplified gene product as a component of the syntaxin 18 complex implicated in Golgi-to-endoplasmic reticulum retrograde transport. 20 2639–2649. 10.1091/mbc.E08-11-1104 19369418 PMC2688544

[B3] Arasaki K. Taniguchi M. Tani K. Tagaya M. (2006). RINT-1 regulates the localization and entry of ZW10 to the syntaxin 18 complex. 17 2780–2788. 10.1091/mbc.e05-10-0973 16571679 PMC1474792

[B4] Arc E. Galland M. Cueff G. Godin B. Lounifi I. Job D. (2011). Reboot the system thanks to protein post-translational modifications and proteome diversity: How quiescent seeds restart their metabolism to prepare seedling establishment. 11 1606–1618. 10.1002/pmic.201000641 21433284

[B5] Bailly C. (2004). Active oxygen species and antioxidants in seed biology. 14 93–107. 10.1079/SSR2004159

[B6] Ballesteros M. Fredriksson A. Henriksson J. Nystrom T. (2001). Bacterial senescence: protein oxidation in non-proliferating cells is dictated by the accuracy of the ribosomes. 20 5280–5289. 10.1093/emboj/20.18.5280 11566891 PMC125621

[B7] Barlowe C. (1997). Coupled ER to Golgi transport reconstituted with purified cytosolic proteins. 139 1097–1108. 10.1083/jcb.139.5.1097 9382859 PMC2140203

[B8] Baskin J. M. Baskin C. C. (2004). A classification system for seed dormancy. 14 1–16. 10.1079/SSR2003150

[B9] Bentsink L. Koornneef M. (2008). Seed dormancy and germination. 6:e0119. 10.1199/tab.0119 22303244 PMC3243337

[B10] Boca S. Koestler F. Ksas B. Chevalier A. Leymarie J. Fekete A. (2014). *Arabidopsis* lipocalins AtCHL and AtTIL have distinct but overlapping functions essential for lipid protection and seed longevity. 37 368–381. 10.1111/pce.12159 23837879

[B11] Bonifacino J. S. Glick B. S. (2004). The mechanisms of vesicle budding and fusion. 116 153–166. 10.1016/S0092-8674(03)01079-1 14744428

[B12] Braakman I. Helenius J. Helenius A. (1992). Manipulating disulfide bond formation and protein folding in the endoplasmic reticulum. 11 1717–1722. 1582407 10.1002/j.1460-2075.1992.tb05223.x PMC556629

[B13] Cao X. Ballew N. Barlowe C. (1998). Initial docking of ER-derived vesicles requires Uso1p and Ypt1p but is independent of SNARE proteins. 17 2156–2165. 10.1093/emboj/17.8.2156 9545229 PMC1170560

[B14] Chatelain E. Hundertmark M. Leprince O. Le Gall S Satour P. Deligny-Penninck S. (2012). Temporal profiling of the heat-stable proteome during late maturation of Medicago truncatula seeds identifies a restricted subset of late embryogenesis abundant proteins associated with longevity. 35 1440–1455. 10.1111/j.1365-3040.2012.02501.x 22380487

[B15] Chen Y. Brandizzi F. (2012). AtIRE1A/AtIRE1B and AGB1 independently control two essential unfolded protein response pathways in Arabidopsis. 69 266–277. 10.1111/j.1365-313X.2011.04788.x 21914012

[B16] Clerkx E. J. M. Vries B. D. Ruys G. J. Groot S. P. C. Koornneef M. (2004). Genetic differences in seed longevity of various *Arabidopsis* mutants. 121 448–461. 10.1111/j.0031-9317.2004.00339.x

[B17] Cutler S. R. Rodriguez P. L. Finkelstein R. R. Abrams S. R. (2010). Abscisic acid: emergence of a core signaling network. 61 651–679. 10.1146/annurev-arplant-042809-112122 20192755

[B18] Dai M. Xue Q. Mccray T. Margavage K. Chen F. Lee J. H. (2013). The PP6 phosphatase regulates ABI5 phosphorylation and abscisic acid signaling in *Arabidopsis*. 25 517–534. 10.1105/tpc.112.105767 23404889 PMC3608775

[B19] Das N. Levine R. L. Orr W. C. Sohal R. S. (2001). Selectivity of protein oxidative damage during aging in *Drosophila melanogaster*. 360 209–216. 10.1042/bj3600209 11696009 PMC1222219

[B20] Ebine K. Okatani Y. Uemura T. Goh T. Shoda K. Niihama M. (2008). A SNARE complex unique to seed plants is required for protein storage vacuole biogenesis and seed development of *Arabidopsis thaliana*. 20 3006–3021. 10.1105/tpc.107.057711 18984676 PMC2613668

[B21] Feng C. Z. Chen Y. Wang C. Kong Y. H. Wu W. H. Chen Y. F. (2014). Arabidopsis RAV1 transcription factor, phosphorylated by SnRK2 kinases, regulates the expression of *ABI3, ABI4*, and *ABI5* during seed germination and early seedling development. 80 654–668. 10.1111/tpj.12670 25231920

[B22] Finkelstein R. R. (1994). Mutations at 2 new *Arabidopsis* ABA response loci are similar to the Abi3 mutations. 5 765–771. 10.1046/j.1365-313X.1994.5060765.x

[B23] Finkelstein R. R. Lynch T. J. (2000). The Arabidopsis abscisic acid response gene *ABI5* encodes a basic leucine zipper transcription factor. 12 599–609. 10.1105/tpc.12.4.599 PMC139856 10760247

[B24] Galland M. Job D. Rajjou L. (2012). The seed proteome web portal. 3:98. 10.3389/fpls.2012.00098 22701460 PMC3371595

[B25] Giurizatto M. I. K. Ferrarese-Filho O. Ferrarese M. D. L. L. Robaina A. D. GonçcAlves M. C. (2012). a-Tocopherol levels in natural and artificial aging of soybean seeds. 34 339–343. 10.4025/actasciagron.v34i3.12660

[B26] Groot S. P. Surki A. A. De Vos R. C. Kodde J. (2012). Seed storage at elevated partial pressure of oxygen, a fast method for analysing seed ageing under dry conditions. 110 1149–1159. 10.1093/aob/mcs198 22967856 PMC3478056

[B27] Guo K. Wang W. Fan W. Wang Z. Zhu M. Tang X. (2018). Arabidopsis GAAP1 and GAAP3 modulate the unfolded protein response and the onset of cell death in response to ER stress. 9:348. 10.3389/fpls.2018.00348 29616060 PMC5864889

[B28] Harman G. E. Mattick L. R. (1976). Association of lipid oxidation with seed ageing and death. 260 323–324. 10.1038/260323a0

[B29] Havaux M. Eymery F. Porfirova S. Rey P. Dormann P. (2005). Vitamin E protects against photoinhibition and photooxidative stress in *Arabidopsis thaliana*. 17 3451–3469. 10.1105/tpc.105.037036 16258032 PMC1315381

[B30] Hetz C. Glimcher L. H. (2009). Fine-tuning of the unfolded protein response: assembling the IRE1alpha interactome. 35 551–561. 10.1016/j.molcel.2009.08.021 19748352 PMC3101568

[B31] Hetz C. Martinon F. Rodriguez D. Glimcher L. H. (2011). The unfolded protein response: integrating stress signals through the stress sensor IRE1alpha. 91 1219–1243. 10.1152/physrev.00001.2011 22013210

[B32] Hirose H. Arasaki K. Dohmae N. Takio K. Hatsuzawa K. Nagahama M. (2004). Implication of ZW10 in membrane trafficking between the endoplasmic reticulum and Golgi. 23 1267–1278. 10.1038/sj.emboj.7600135 15029241 PMC381410

[B33] Hu Y. Yu D. (2014). BRASSINOSTEROID INSENSITIVE2 interacts with ABSCISIC ACID INSENSITIVE5 to mediate the antagonism of brassinosteroids to abscisic acid during seed germination in *Arabidopsis*. 26 4394–4408. 10.1105/tpc.114.130849 25415975 PMC4277219

[B34] Huang X. Zhang X. Gong Z. Yang S. Shi Y. (2017). ABI4 represses the expression of type-A *ARRs* to inhibit seed germination in Arabidopsis. 89 354–365. 10.1111/tpj.13389 27711992

[B35] Iwata Y. Fedoroff N. V. Koizumi N. (2008). Arabidopsis bZIP60 is a proteolysis-activated transcription factor involved in the endoplasmic reticulum stress response. 20 3107–3121. 10.1105/tpc.108.061002 19017746 PMC2613661

[B36] Iwata Y. Koizumi N. (2005). An Arabidopsis transcription factor, AtbZIP60, regulates the endoplasmic reticulum stress response in a manner unique to plants. 102 5280–5285. 10.1073/pnas.0408941102 15781873 PMC555978

[B37] Iwata Y. Nishino T. Takayama S. Koizumi N. (2010a). Characterization of a plant-specific gene induced by endoplasmic reticulum stress in *Arabidopsis thaliana*. 74 2087–2091. 20944397 10.1271/bbb.100487

[B38] Iwata Y. Sakiyama M. Lee M.-H. Koizumi N. (2010b). Transcriptomic response of *Arabidopsis thaliana* to tunicamycin induced endoplasmic reticulum stress. 27 161–171.

[B39] Jahn R. Scheller R. H. (2006). SNAREs–engines for membrane fusion. 7 631–643. 10.1038/nrm2002 16912714

[B40] Jeevan Kumar S. P. Rajendra Prasad S. Banerjee R. Thammineni C. (2015). Seed birth to death: dual functions of reactive oxygen species in seed physiology. 116 663–668. 10.1093/aob/mcv098 26271119 PMC4578000

[B41] Job C. Rajjou L. Lovigny Y. Belghazi M. Job D. (2005). Patterns of protein oxidation in Arabidopsis seeds during germination. 138 790–802. 10.1104/pp.105.062778 15908592 PMC1150397

[B42] Johansson E. Olsson O. Nystrom T. (2004). Progression and specificity of protein oxidation in the life cycle of *Arabidopsis thaliana*. 279 22204–22208. 10.1074/jbc.M402652200 15070894

[B43] Kadowaki H. Nishitoh H. Ichijo H. (2004). Survival and apoptosis signals in ER stress: the role of protein kinases. 28 93–100. 10.1016/j.jchemneu.2004.05.004 15363494

[B44] Kalemba E. M. Pukacka S. (2014). Carbonylated proteins accumulated as vitality decreases during long-term storage of beech (*Fags sylvatica* L.) seeds. 28 503–515. 10.1007/s00468-013-0967-9

[B45] Kanai M. Nishimura M. Hayashi M. (2010). A peroxisomal ABC transporter promotes seed germination by inducing pectin degradation under the control of *ABI5*. 62 936–947. 10.1111/j.1365-313X.2010.04205.x 20345608

[B46] Koizumi N. (1996). Isolation and responses to stress of a gene that encodes a luminal binding protein in *Arabidopsis thaliana*. 37 862–865. 10.1093/oxfordjournals.pcp.a029023 8888624

[B47] Korolainen M. A. Goldsteins G. Alafuzoff I. Koistinaho J. Pirttila T. (2002). Proteomic analysis of protein oxidation in Alzheimer’s disease brain. 23 3428–3433. 10.1002/1522-2683(200210)23:19<3428::AID-ELPS3428>3.0.CO;2-5 12373773

[B48] Kraynack B. A. Chan A. Rosenthal E. Essid M. Umansky B. Waters M. G. (2005). Dsl1p, Tip20p, and the novel Dsl3(Sec39) protein are required for the stability of the Q/t-SNARE complex at the endoplasmic reticulum in yeast. 16 3963–3977. 10.1091/mbc.e05-01-0056 15958492 PMC1196311

[B49] Lee M. C. Miller E. A. Goldberg J. Orci L. Schekman R. (2004). Bi-directional protein transport between the ER and Golgi. 20 87–123. 10.1146/annurev.cellbio.20.010403.105307 15473836

[B50] Li L. Shimada T. Takahashi H. Koumoto Y. Shirakawa M. Takagi J. (2013). MAG2 and three MAG2-INTERACTING PROTEINs form an ER-localized complex to facilitate storage protein transport in *Arabidopsis thaliana*. 76 781–791. 10.1111/tpj.12347 24118572

[B51] Li L. Shimada T. Takahashi H. Ueda H. Fukao Y. Kondo M. (2006). MAIGO2 is involved in exit of seed storage proteins from the endoplasmic reticulum in *Arabidopsis thaliana*. 18 3535–3547. 10.1105/tpc.106.046151 17194767 PMC1785406

[B52] Liu J. X. Howell S. H. (2010). bZIP28 and NF-Y transcription factors are activated by ER stress and assemble into a transcriptional complex to regulate stress response genes in *Arabidopsis*. 22 782–796. 10.1105/tpc.109.072173 20207753 PMC2861475

[B53] Liu J. X. Srivastava R. Che P. Howell S. H. (2007). An endoplasmic reticulum stress response in *Arabidopsis* is mediated by proteolytic processing and nuclear relocation of a membrane-associated transcription factor, bZIP28. 19 4111–4119. 10.1105/tpc.106.050021 18156219 PMC2217655

[B54] Lois L. M. Lima C. D. Chua N. H. (2003). Small ubiquitin-like modifier modulates abscisic acid signaling in Arabidopsis. 15 1347–1359. 10.1105/tpc.009902 12782728 PMC156371

[B55] Long R. L. Gorecki M. J. Renton M. Scott J. K. Colville L. Goggin D. E. (2015). The ecophysiology of seed persistence: a mechanistic view of the journey to germination or demise. 90 31–59. 10.1111/brv.12095 24618017

[B56] Lopez-Molina L. Mongrand S. McLachlin D. T. Chait B. T. Chua N. H. (2002). ABI5 acts downstream of ABI3 to execute an ABA-dependent growth arrest during germination. 32 317–328. 10.1046/j.1365-313X.2002.01430.x 12410810

[B57] Ma Y. Szostkiewicz I. Korte A. Moes D. Yang Y. Christmann A. (2009). Regulators of PP2C phosphatase activity function as abscisic acid sensors. 324 1064–1068. 10.1126/science.1172408 19407143

[B58] Marciniak S. J. Ron D. (2006). Endoplasmic reticulum stress signaling in disease. 86 1133–1149. 10.1152/physrev.00015.2006 17015486

[B59] Martin R. C. Liu P. P. Nonogaki H. (2005). Simple purification of small RNAs from seeds efficient detection of multiple microRNAs expressed in *Arabidopsis thaliana* and tomato (*Lycopersicon esculentum*) seeds. 15 319–328. 10.1079/SSR2005220

[B60] Martinez I. M. Chrispeels M. J. (2003). Genomic analysis of the unfolded protein response in Arabidopsis shows its connection to important cellular processes. 15 561–576. 10.1105/tpc.007609 12566592 PMC141221

[B61] Miura K. Hasegawa P. M. (2010). Sumoylation and other ubiquitin-like post-translational modifications in plants. 20 223–232. 10.1016/j.tcb.2010.01.007 20189809

[B62] Miura K. Lee J. Jin J. B. Yoo C. Y. Miura T. Hasegawa P. M. (2009). Sumoylation of ABI5 by the *Arabidopsis* SUMO E3 ligase SIZ1 negatively regulates abscisic acid signaling. 106 5418–5423. 10.1073/pnas.0811088106 19276109 PMC2664011

[B63] Mostertz J. Hecker M. (2003). Patterns of protein carbonylation following oxidative stress in wild-type and sigB *Bacillus subtilis* cells. 269 640–648. 10.1007/s00438-003-0877-4 12845527

[B64] Murthy U. M. Sun W. Q. (2000). Protein modification by Amadori and Maillard reactions during seed storage: roles of sugar hydrolysis and lipid peroxidation. 51 1221–1228. 10.1093/jexbot/51.348.1221 10937697

[B65] Nagashima Y. Mishiba K. Suzuki E. Shimada Y. Iwata Y. Koizumi N. (2011). Arabidopsis IRE1 catalyses unconventional splicing of bZIP60 mRNA to produce the active transcription factor. 1:29. 10.1038/srep00029 22355548 PMC3216516

[B66] Nakashima K. Fujita Y. Kanamori N. Katagiri T. Umezawa T. Kidokoro S. (2009). Three Arabidopsis SnRK2 protein kinases, SRK2D/SnRK2.2, SRK2E/SnRK2.6/OST1 and SRK2I/SnRK2.3, involved in ABA signaling are essential for the control of seed development and dormancy. 50 1345–1363. 10.1093/pcp/pcp083 19541597

[B67] Nguyen T. P. Cueff G. Hegedus D. D. Rajjou L. Bentsink L. (2015). A role for seed storage proteins in *Arabidopsis* seed longevity. 66 6399–6413. 10.1093/jxb/erv348 26184996 PMC4588887

[B68] Noh S. J. Kwon C. S. Oh D. H. Moon J. S. Chung W. I. (2003). Expression of an evolutionarily distinct novel BiP gene during the unfolded protein response in *Arabidopsis thaliana*. 311 81–91. 10.1016/S0378-1119(03)00559-6 12853141

[B69] North H. Baud S. Debeaujon I. Dubos C. Dubreucq B. Grappin P. (2010). Arabidopsis seed secrets unravelled after a decade of genetic and omics-driven research. 61 971–981. 10.1111/j.1365-313X.2009.04095.x 20409271

[B70] Ooms J. Leon-Kloosterziel K. M. Bartels D. Koornneef M. Karssen C. M. (1993). Acquisition of desiccation tolerance and longevity in seeds of *Arabidopsis thaliana* (a comparative study using abscisic acid-insensitive abi3 mutants). 102 1185–1191. 10.1104/pp.102.4.1185 12231895 PMC158904

[B71] Piskurewicz U. Jikumaru Y. Kinoshita N. Nambara E. Kamiya Y. Lopez-Molina L. (2008). The gibberellic acid signaling repressor RGL2 inhibits *Arabidopsis* seed germination by stimulating abscisic acid synthesis and ABI5 activity. 20 2729–2745. 10.1105/tpc.108.061515 18941053 PMC2590721

[B72] Prieto-Dapena P. Castano R. Almoguera C. Jordano J. (2006). Improved resistance to controlled deterioration in transgenic seeds. 142 1102–1112. 10.1104/pp.106.087817 16998084 PMC1630740

[B73] Rajjou L. Lovigny Y. Groot S. P. Belghazi M. Job C. Job D. (2008). Proteome-wide characterization of seed aging in Arabidopsis: a comparison between artificial and natural aging protocols. 148 620–641. 10.1104/pp.108.123141 18599647 PMC2528126

[B74] Ren Y. Yip C. K. Tripathi A. Huie D. Jeffrey P. D. Walz T. (2009). A structure-based mechanism for vesicle capture by the multisubunit tethering complex Dsl1. 139 1119–1129. 10.1016/j.cell.2009.11.002 20005805 PMC2806190

[B75] Sano N. Rajjou L. North H. M. Debeaujon I. Marion-Poll A. Seo M. (2016). Staying alive: molecular aspects of seed longevity. 57 660–674. 10.1093/pcp/pcv186 26637538

[B76] Sattler S. E. Gilliland L. U. Magallanes-Lundback M. Pollard M. Dellapenna D. (2004). Vitamin E is essential for seed longevity and for preventing lipid peroxidation during germination. 16 1419–1432. 10.1105/tpc.021360 15155886 PMC490036

[B77] Schmitt H. D. (2010). Dsl1p/Zw10: common mechanisms behind tethering vesicles and microtubules. 20 257–268. 10.1016/j.tcb.2010.02.001 20226673

[B78] Shimada T. Fuji K. Tamura K. Kondo M. Nishimura M. Hara-Nishimura I. (2003). Vacuolar sorting receptor for seed storage proteins in *Arabidopsis thaliana*. 100 16095–16100. 10.1073/pnas.2530568100 14657332 PMC307698

[B79] Shkolnik-Inbar D. Bar-Zvi D. (2010). ABI4 mediates abscisic acid and cytokinin inhibition of lateral root formation by reducing polar auxin transport in *Arabidopsis*. 22 3560–3573. 10.1105/tpc.110.074641 21097710 PMC3015119

[B80] Shu K. Chen Q. Wu Y. Liu R. Zhang H. Wang P. (2016a). ABI4 mediates antagonistic effects of abscisic acid and gibberellins at transcript and protein levels. 85 348–361. 10.1111/tpj.13109 26708041

[B81] Shu K. Chen Q. Wu Y. Liu R. Zhang H. Wang S. (2016b). ABSCISIC ACID-INSENSITIVE 4 negatively regulates flowering through directly promoting Arabidopsis *FLOWERING LOCUS C* transcription. 67 195–205. 10.1093/jxb/erv459 26507894 PMC4682436

[B82] Shu K. Liu X. D. Xie Q. He Z. H. (2016c). Two faces of one seed: hormonal regulation of dormancy and germination. 9 34–45. 10.1016/j.molp.2015.08.010 26343970

[B83] Shu K. Zhang H. Wang S. Chen M. Wu Y. Tang S. (2013). ABI4 regulates primary seed dormancy by regulating the biogenesis of abscisic acid and gibberellins in Arabidopsis. 9:e1003577. 10.1371/journal.pgen.1003577 23818868 PMC3688486

[B84] Shu K. Zhou W. Yang W. (2018). APETALA 2-domain-containing transcription factors: focusing on abscisic acid and gibberellins antagonism. 217 977–983. 10.1111/nph.14880 29058311

[B85] Soderman E. M. Brocard I. M. Lynch T. J. Finkelstein R. R. (2000). Regulation and function of the Arabidopsis *ABA-insensitive4* gene in seed and abscisic acid response signaling networks. 124 1752–1765. 10.1104/pp.124.4.1752 PMC59872 11115891

[B86] Surpin M. Raikhel N. (2004). Traffic jams affect plant development and signal transduction. 5 100–109. 10.1038/nrm1311 15040443

[B87] Tajima H. Iwata Y. Iwano M. Takayama S. Koizumi N. (2008). Identification of an *Arabidopsis* transmembrane bZIP transcription factor involved in the endoplasmic reticulum stress response. 374 242–247. 10.1016/j.bbrc.2008.07.021 18634751

[B88] Talent J. M. Kong Y. Gracy R. W. (1998). A double stain for total and oxidized proteins from two-dimensional fingerprints. 263 31–38. 10.1006/abio.1998.2752 9750139

[B89] Tripathi A. Ren Y. Jeffrey P. D. Hughson F. M. (2009). Structural characterization of Tip20p and Dsl1p, subunits of the Dsl1p vesicle tethering complex. 16 114–123. 10.1038/nsmb.1548 19151722 PMC2635920

[B90] Uemura T. Sato T. Aoki T. Yamamoto A. Okada T. Hirai R. (2009). p31 deficiency influences endoplasmic reticulum tubular morphology and cell survival. 29 1869–1881. 10.1128/MCB.01089-08 19188447 PMC2655616

[B91] Vaistij F. E. Gan Y. Penfield S. Gilday A. D. Dave A. He Z. (2013). Differential control of seed primary dormancy in *Arabidopsis* ecotypes by the transcription factor SPATULA. 110 10866–10871. 10.1073/pnas.1301647110 23754415 PMC3696787

[B92] Vukasinovic N. Zarsky V. (2016). Tethering complexes in the Arabidopsis endomembrane system. 4:46 10.3389/fcell.2016.00046 PMC4871884 27243010

[B93] Walters C. (1998). Understanding the mechanisms and kinetics of seed aging. 8 223–244. 10.1016/j.plaphy.2012.07.031 22995217

[B94] Whyte J. R. Munro S. (2002). Vesicle tethering complexes in membrane traffic. 115 2627–2637. 10.1242/jcs.115.13.2627 12077354

[B95] Wilson D. O. McDonald M. B. (1986). The lipid peroxidation model of seed aging. 14 269–300.

[B96] Xiao J. Liu C. C. Chen P. L. Lee W. H. (2001). RINT-1, a novel Rad50-interacting protein, participates in radiation-induced G_2_/M checkpoint control. 276 6105–6111. 10.1074/jbc.M008893200 11096100

[B97] Yoshida H. (2007). ER stress and diseases. 274 630–658. 10.1111/j.1742-4658.2007.05639.x 17288551

[B98] Yu F. Wu Y. Xie Q. (2015). Precise protein post-translational modifications modulate ABI5 activity. 20 569–575. 10.1016/j.tplants.2015.05.004 26044742

[B99] Yu I. M. Hughson F. M. (2010). Tethering factors as organizers of intracellular vesicular traffic. 26 137–156. 10.1146/annurev.cellbio.042308.113327 19575650

[B100] Zhao P. Liu F. Zhang B. Liu X. Wang B. Gong J. (2013). MAIGO2 is involved in abscisic acid-mediated response to abiotic stresses and Golgi-to-ER retrograde transport. 148 246–260. 10.1111/j.1399-3054.2012.01704.x 23025793

