# Supplementary material for: Misregulation of ER-Golgi Vesicle Transport Induces ER Stress and Affects Seed Vigor and Stress Response
Source: Front Plant Sci. 2018 May 18;9:658. doi: 10.3389/fpls.2018.00658 (PMC5968616; doi:10.3389/fpls.2018.00658)
Supplement: Supplementary file 1 [file Presentation_1.pptx]

## Slide 1
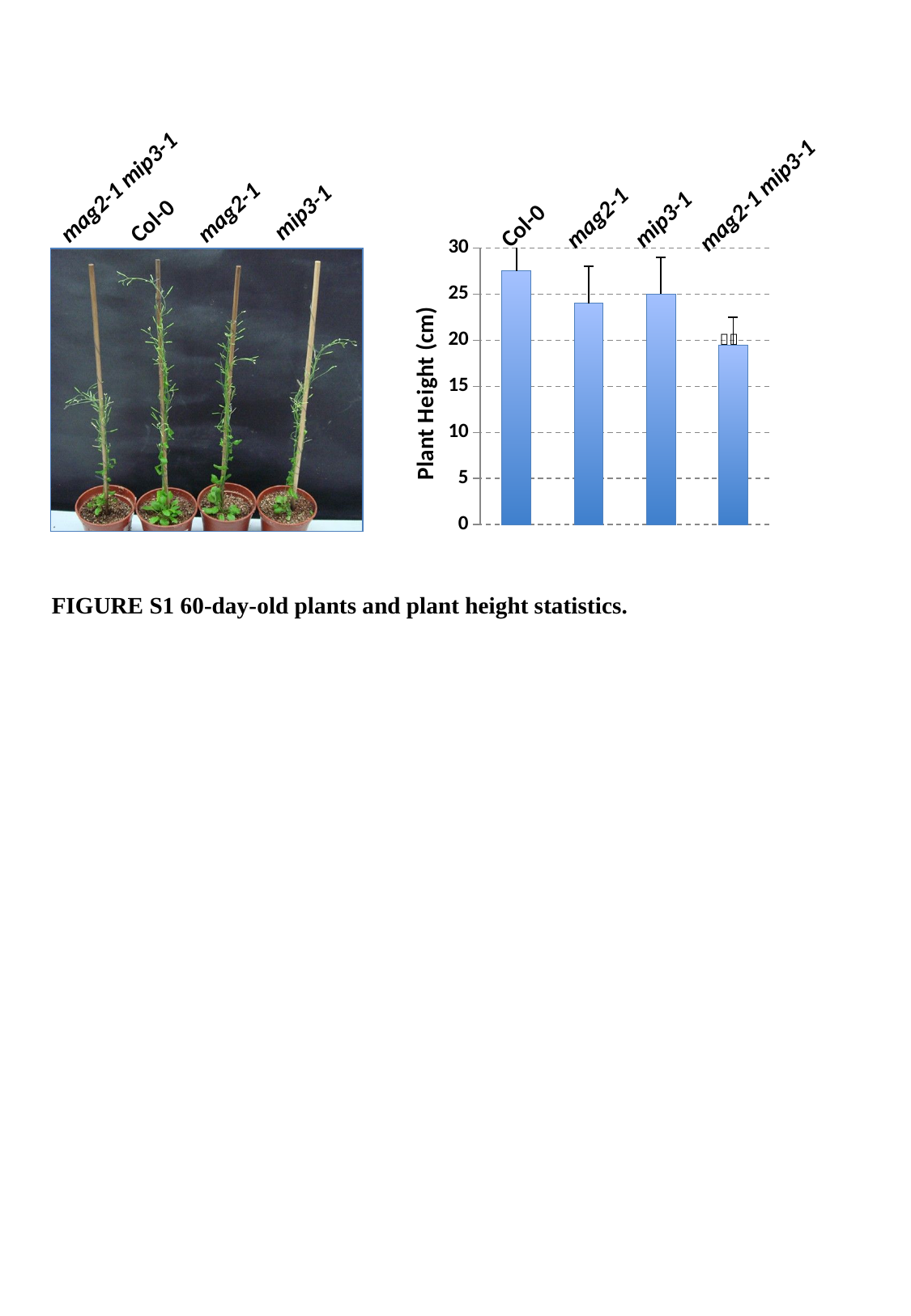

mip3-1
mag2-1 mip3-1
mag2-1
Col-0
mag2-1 mip3-1
mag2-1
mip3-1
Col-0
### Chart
| Category | |
|---|---|
| Col-0 | 27.5 |
| mag2-1 | 24.0 |
| mip3-1 | 25.0 |
| mag2-1 mip3-1 | 19.5 |＊
＊
FIGURE S1 60-day-old plants and plant height statistics.

## Slide 2
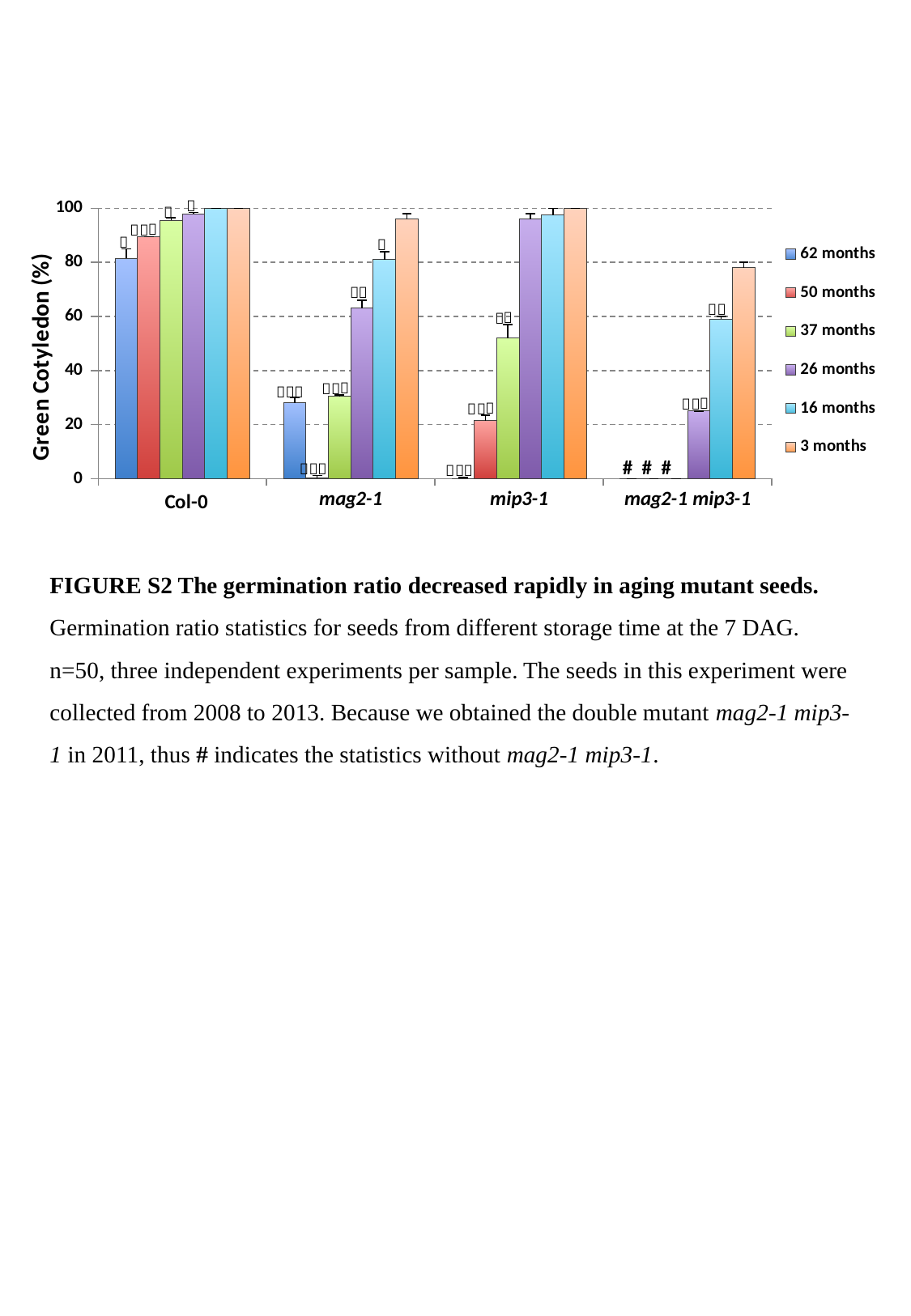

### Chart
| Category | 62 months | 50 months | 37 months | 26 months | 16 months | 3 months |
|---|---|---|---|---|---|---|
| Col-0 | 81.5 | 89.5 | 95.5 | 98.0 | 100.0 | 100.0 |
| mag2-1 | 28.0 | 0.15 | 30.5 | 63.0 | 81.0 | 96.0 |
| mip3-1 | 0.0 | 21.5 | 52.0 | 96.0 | 97.5 | 100.0 |
| mag2-1 mip3-1 | 0.0 | 0.0 | 0.0 | 25.0 | 59.0 | 78.0 |# # #
＊
＊
＊
＊
＊
＊
＊
＊
＊
＊
＊
＊
＊
＊
＊
＊
＊
＊
＊
＊
＊
＊
＊
＊
＊
＊
＊
＊
＊
＊
＊
FIGURE S2 The germination ratio decreased rapidly in aging mutant seeds.
Germination ratio statistics for seeds from different storage time at the 7 DAG. n=50, three independent experiments per sample. The seeds in this experiment were collected from 2008 to 2013. Because we obtained the double mutant mag2-1 mip3-1 in 2011, thus # indicates the statistics without mag2-1 mip3-1.

## Slide 3
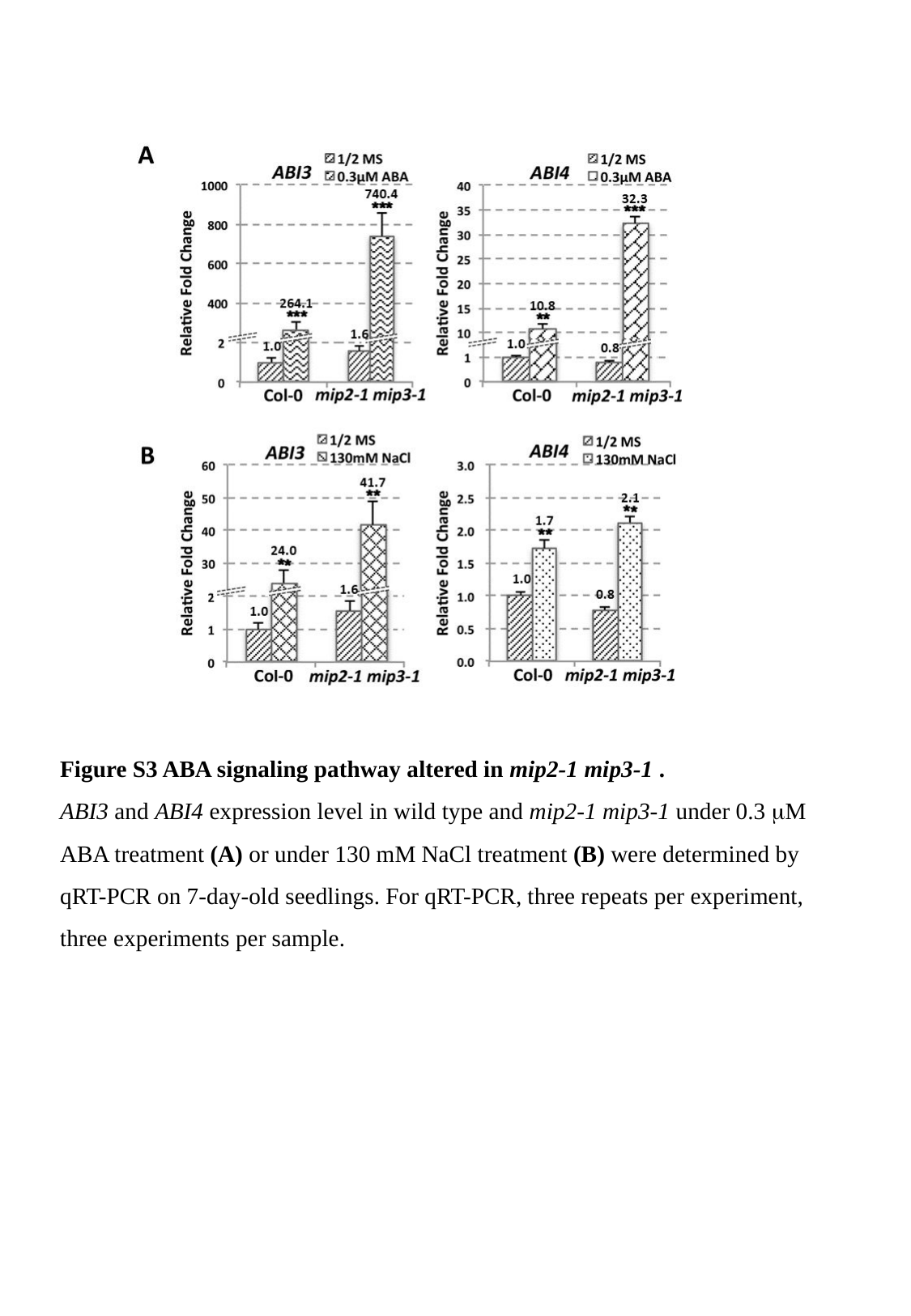

Figure S3 ABA signaling pathway altered in mip2-1 mip3-1 .
ABI3 and ABI4 expression level in wild type and mip2-1 mip3-1 under 0.3 mM ABA treatment (A) or under 130 mM NaCl treatment (B) were determined by qRT-PCR on 7-day-old seedlings. For qRT-PCR, three repeats per experiment, three experiments per sample.

## Slide 4
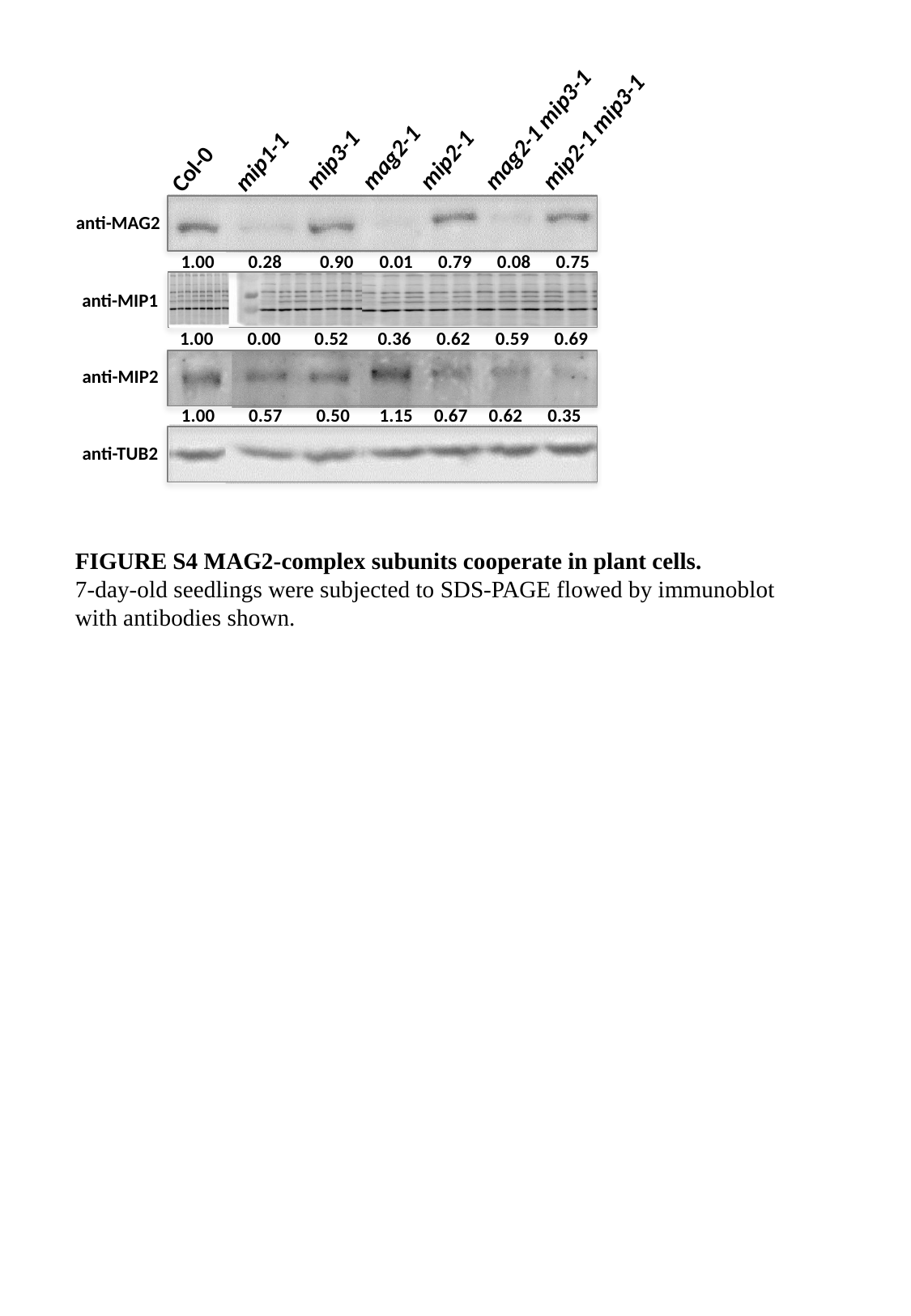

mag2-1 mip3-1
mip2-1 mip3-1
mag2-1
mip3-1
mip1-1
mip2-1
Col-0
anti-MAG2
 1.00 0.28 0.90 0.01 0.79 0.08 0.75
anti-MIP1
1.00 0.00 0.52 0.36 0.62 0.59 0.69
anti-MIP2
1.00 0.57 0.50 1.15 0.67 0.62 0.35
anti-TUB2
FIGURE S4 MAG2-complex subunits cooperate in plant cells.
7-day-old seedlings were subjected to SDS-PAGE flowed by immunoblot with antibodies shown.

## Slide 5
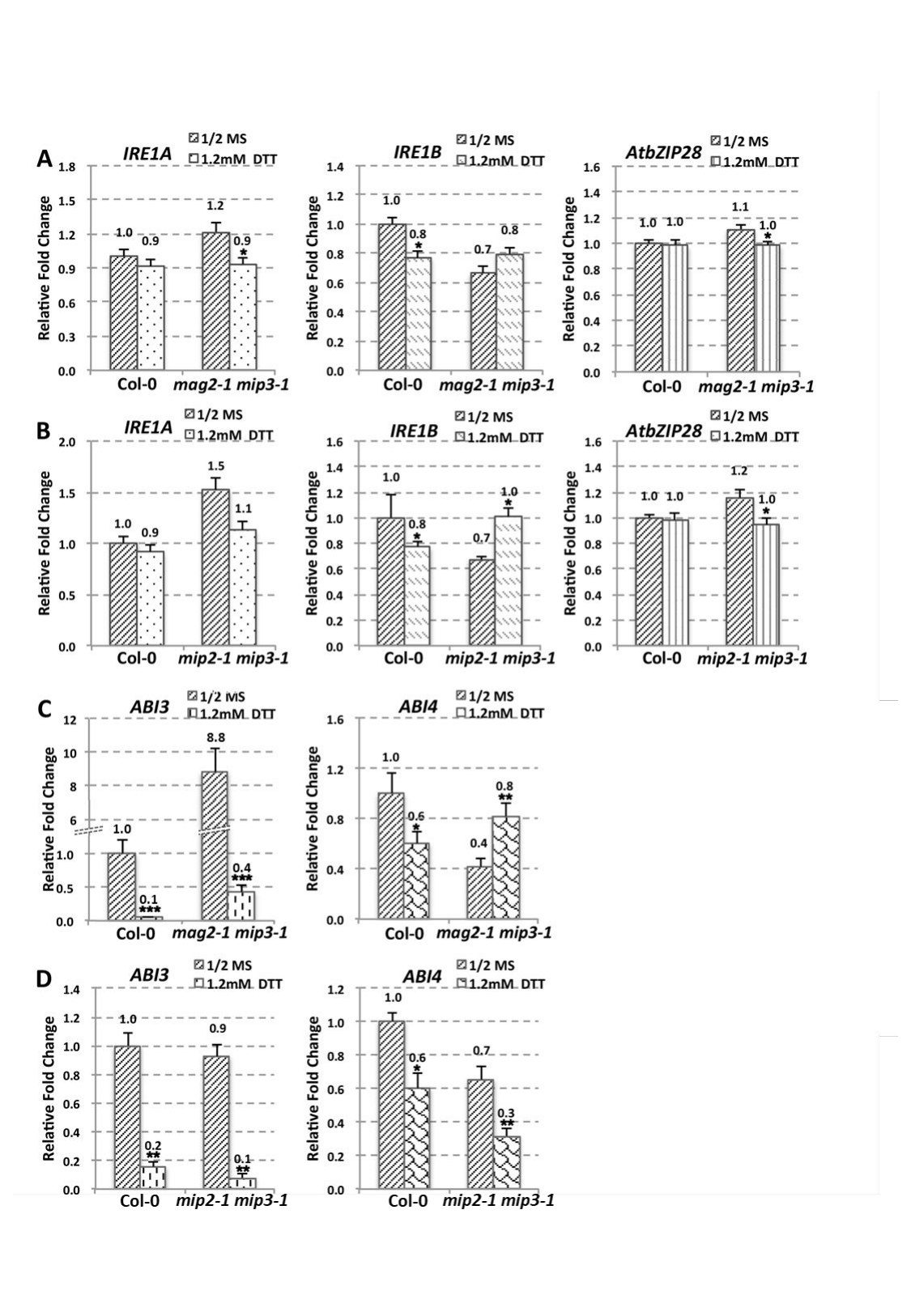

## Slide 6
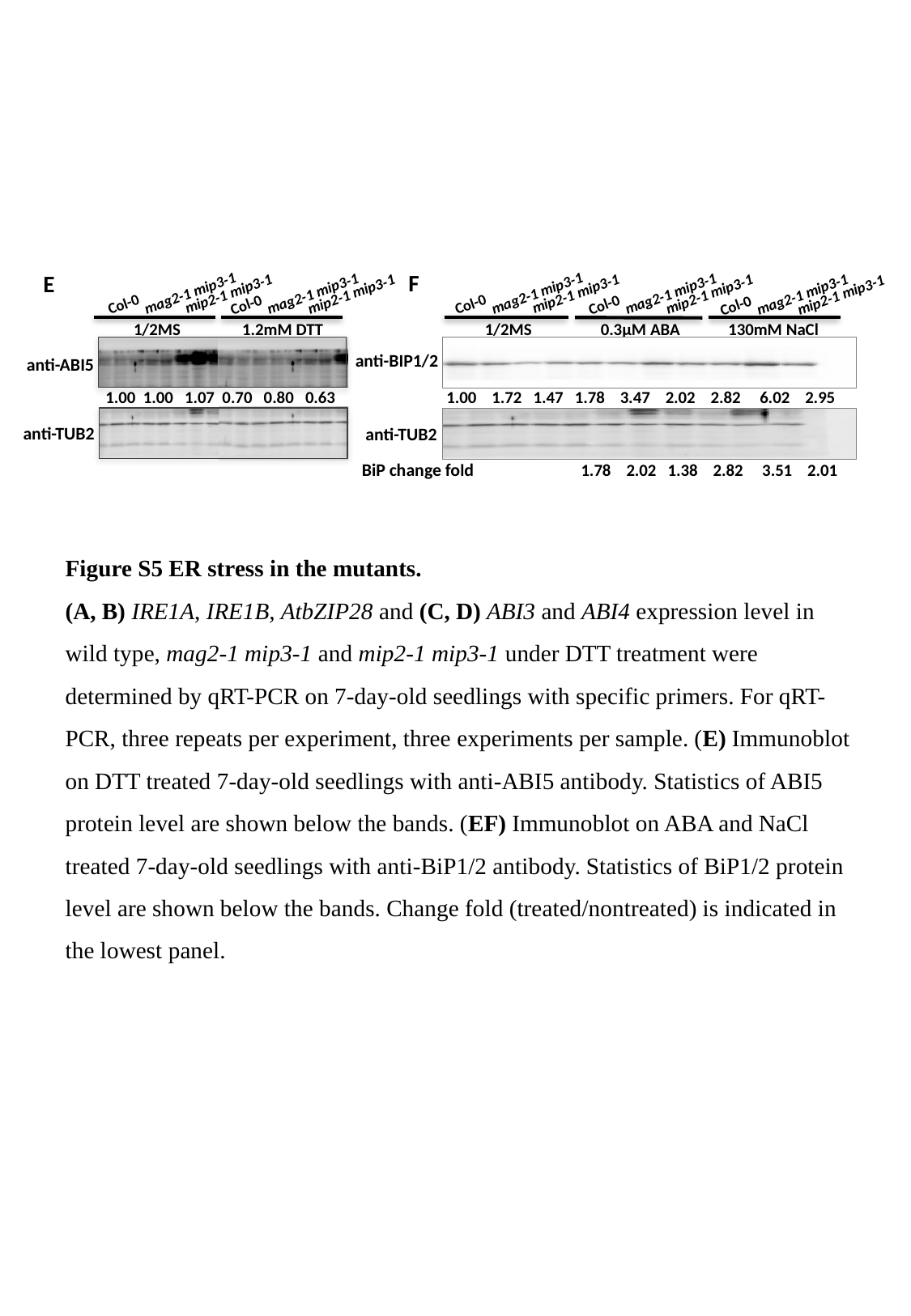

F
E
mag2-1 mip3-1
mip2-1 mip3-1
Col-0
mag2-1 mip3-1
mip2-1 mip3-1
Col-0
1/2MS
1.2mM DTT
anti-ABI5
1.00 1.00 1.07 0.70 0.80 0.63
anti-TUB2
mag2-1 mip3-1
mip2-1 mip3-1
Col-0
mag2-1 mip3-1
mip2-1 mip3-1
Col-0
mag2-1 mip3-1
mip2-1 mip3-1
Col-0
0.3μM ABA
1/2MS
130mM NaCl
anti-BIP1/2
1.00 1.72 1.47 1.78 3.47 2.02 2.82 6.02 2.95
anti-TUB2
BiP change fold 1.78 2.02 1.38 2.82 3.51 2.01
Figure S5 ER stress in the mutants.
(A, B) IRE1A, IRE1B, AtbZIP28 and (C, D) ABI3 and ABI4 expression level in wild type, mag2-1 mip3-1 and mip2-1 mip3-1 under DTT treatment were determined by qRT-PCR on 7-day-old seedlings with specific primers. For qRT-PCR, three repeats per experiment, three experiments per sample. (E) Immunoblot on DTT treated 7-day-old seedlings with anti-ABI5 antibody. Statistics of ABI5 protein level are shown below the bands. (EF) Immunoblot on ABA and NaCl treated 7-day-old seedlings with anti-BiP1/2 antibody. Statistics of BiP1/2 protein level are shown below the bands. Change fold (treated/nontreated) is indicated in the lowest panel.
